# A Multi-Task Deep Learning Method for Detection of Meniscal Tears in MRI Data from the Osteoarthritis Initiative Database

**DOI:** 10.3389/fbioe.2021.747217

**Published:** 2021-12-02

**Authors:** Alexander Tack, Alexey Shestakov, David Lüdke, Stefan Zachow

**Affiliations:** ^1^ Dept. for Visual and Data-Centric Computing, Zuse Institute Berlin, Berlin, Germany; ^2^ Charité–University Medicine, Berlin, Germany

**Keywords:** knee joint, meniscal lesions, convolutional neural networks–CNN, residual learning, explainable AI (XAI), multi-task deep learning, bounding box regression, object detection

## Abstract

We present a novel and computationally efficient method for the detection of meniscal tears in Magnetic Resonance Imaging (MRI) data. Our method is based on a Convolutional Neural Network (CNN) that operates on complete 3D MRI scans. Our approach detects the presence of meniscal tears in three anatomical sub-regions (anterior horn, body, posterior horn) for both the Medial Meniscus (MM) and the Lateral Meniscus (LM) individually. For optimal performance of our method, we investigate how to preprocess the MRI data and how to train the CNN such that only relevant information within a Region of Interest (RoI) of the data volume is taken into account for meniscal tear detection. We propose meniscal tear detection combined with a bounding box regressor in a multi-task deep learning framework to let the CNN implicitly consider the corresponding RoIs of the menisci. We evaluate the accuracy of our CNN-based meniscal tear detection approach on 2,399 Double Echo Steady-State (DESS) MRI scans from the Osteoarthritis Initiative database. In addition, to show that our method is capable of generalizing to other MRI sequences, we also adapt our model to Intermediate-Weighted Turbo Spin-Echo (IW TSE) MRI scans. To judge the quality of our approaches, Receiver Operating Characteristic (ROC) curves and Area Under the Curve (AUC) values are evaluated for both MRI sequences. For the detection of tears in DESS MRI, our method reaches AUC values of 0.94, 0.93, 0.93 (anterior horn, body, posterior horn) in MM and 0.96, 0.94, 0.91 in LM. For the detection of tears in IW TSE MRI data, our method yields AUC values of 0.84, 0.88, 0.86 in MM and 0.95, 0.91, 0.90 in LM. In conclusion, the presented method achieves high accuracy for detecting meniscal tears in both DESS and IW TSE MRI data. Furthermore, our method can be easily trained and applied to other MRI sequences.

## 1 Introduction

Menisci are hydrated fibrocartilaginous soft tissues within the knee joint that absorb shocks, provide lubrication, and allow for joint stability during movement (Markes et al., 2020). In patients with symptomatic osteoarthritis, meniscal damage is also found very frequently with a prevalence of up to 91% (Bhattacharyya et al., 2003). Meniscal tears are usually caused by trauma and degeneration (Beaufils and Pujol, 2017) and might lead to a loss of function, early osteoarthritis, tibiofemoral osteophytes, and cartilage loss (Ding et al., 2007; Snoeker et al., 2021). Magnetic Resonance Imaging (MRI) is commonly used for the noninvasive assessment of meniscal morphology since MRI provides a three-dimensional view of the knee joint with high contrast between soft tissues. Hence, MRI is the recognized screening tool for diagnostic assessment before performing therapeutic arthroscopy or any other treatment (Crawford et al., 2007). Among other factors, a proper treatment concept for meniscal damage depends highly on the type of tear and its location (Englund et al., 2001; Beaufils and Pujol, 2017). An appropriate medical intervention can delay further development of arthritic changes, improve quality of life, and reduce healthcare expenditures. However, in practice, the optimal treatment is not always apparent (Khan et al., 2014; Kise et al., 2016), while an improper procedure might even lead to an acceleration of osteoarthritis progression (Roemer et al., 2017). For this reason, an accurate and reliable diagnosis of meniscal tears in view of their location, type, and orientation is important.

The diagnosis of meniscal tears in MRI is a time consuming and tedious procedure. These defects are often difficult to detect due to their small sizes and arbitrary orientations. It is frequently necessary to go back and forth in the MRI slices and switch view directions for a thorough assessment of occurrences and spatial extents of pathological changes. In addition, the meniscal representation in the image data depends on the chosen MRI sequence. What appears clearly visible in one sequence may be barely noticeable in another due to insufficient contrast. Computer-Aided Diagnosis (CAD) attempts to overcome some of these limitations. CAD tools can be employed to increase the sensitivity and specificity of physicians in detecting and classifying meniscal tears (Bien et al., 2018; Pedoia et al., 2019; Kunze et al., 2020). Moreover, CAD could speed up the diagnosis, reduce the number of unintentionally missed defects, avoid unnecessary interventions (e.g., arthroscopic interventions), and lead to fewer treatment delays. Several CAD approaches for an automated detection of meniscal tears in MRI data have been proposed in recent years. A distinction can be made between methods that evaluate the 2D contents of cross-sectional images often coming from a set of curated slices (2D approaches) and those that evaluate 3D image information in the MRI data volume (3D approaches). In the context of image analysis by means of Convolutional Neural Networks (CNNs), we distinguish between 2D CNNs and 3D CNNs. In the case of the 2D approaches, there exists a pseudo-3D variant in which sets of (neighboring) sectional images are included in the evaluation. In these pseudo-3D variants, 2D CNNs are employed to encode 2D slices of a 3D MRI dataset. Afterwards, the respective 2D encodings are condensed (e.g., by global max- or average-pooling), concatenated, and passed to a classifier.

Roblot et al. (2019) proposed a method to detect meniscal tears from a curated set of sagittal 2D MRI slices. Their approach is based on the 2D “faster R-CNN” (Ren et al., 2015) and comprises three steps: Firstly, the positions of both meniscal horns are detected; secondly, the presence of a tear is classified; and thirdly, the respective tear orientation is determined. The method yields an Area Under the Curve (AUC) of the Receiver Operating Characteristic (ROC) of 0.92 for the detection of the meniscal horns’ positions, an AUC of 0.94 for detecting the presence of meniscal tears, and an AUC of 0.83 for the determination of the tear orientations. Couteaux et al. (2019) presented a similar method, also detecting meniscal tears from a curated set of sagittal 2D MRI slices. They employed a masked region-based 2D CNN (He et al., 2017) to locate the anterior and the posterior horns of the Medial Meniscus (MM) as well as the Lateral Meniscus (LM). Their method yields on average an AUC of 0.906 for all three tasks, i.e. the location of the respective region, the detection of meniscal tears, and the classification of the tear orientation.

Processing of all MRI slices instead of individually selected ones was performed by Bien et al. (2018) who proposed a 2D CNN for the detection of meniscal tears. Their method achieves an AUC of 0.847. Pedoia et al. (2019) adopted a method that combined a 2D CNN for meniscus segmentation with a 3D CNN for detection and severity assessment of meniscal tears. This approach was able to differentiate between tears and no tears with an AUC of 0.89. Tsai et al. (2020) proposed a so-called “Efficiently-Layered Network” for detection of meniscal tears, reaching an AUC of 0.904 and 0.913 for two different datasets. Azcona et al. (2020) demonstrated the use of a 2D CNN as a pseudo-3D variant for detection of torn menisci. Their method relies on transfer learning while using data augmentation and reaches an AUC of 0.934. Fritz et al. (2020) presented a deep 3D CNN to detect tears in MRI data for MM and LM, respectively. Their method reaches AUC values of 0.882, 0.781, and 0.961 for the detection of medial, lateral, and overall meniscal tears. Rizk et al. (2021) also proposed a 3D CNN for meniscal tear detection in MRI data for MM and LM individually. Their approach yields an AUC of 0.93 for MM and 0.84 for LM.

A common limitation among many of the methods listed above is their strong reliance on segmentations of the menisci (or at least of bounding boxes), which can be challenging to obtain due to the inhomogeneous appearance of pathological menisci in MRI data as well as an insufficient contrast to adjacent tissues (Rahman et al., 2020). Furthermore, some approaches merely operate on 2D slices. A major limitation of such methods is that the trained 2D CNNs cannot take whole MRI volumes into account, thus possibly missing important feature correlations in 3D space. Besides, an appropriate selection of curated slices requires expert knowledge. Therefore, the applicability of these methods to 3D volumes is unclear since they were not trained on 3D data. Finally, none of the presented methods is able to detect meniscal tears for all anatomical sub-regions of the menisci individually, i.e., the anterior horn, the meniscal body, and the posterior horn.

Our motivation is to detect meniscal tears in MRI data more accurately than previous methods in terms of correctness and localization. For this purpose, we present a method that detects tears in anatomical sub-regions of both the MM and the LM. We design our study in a manner that allows for a comparison of different possible approaches. Moreover, the study shows our progression in addressing the task of meniscal tear detection in 3D MR images. We investigate how to handle best the input data such that the least pre-processing is required for inference and the best accuracy is achieved. Furthermore, we show that our proposed method generalizes well to different MRI sequences. We employ two ResNet architectures (He et al., 2016; Yu et al., 2017) to classify meniscal tears in each sub-region of the MM and the LM, respectively, utilizing three different approaches.

In a first approach (i), we train a 3D CNN on the complete 3D MRI dataset as input. We call it *Full-scale* approach within the remainder of this article.

Since large input data requires a lot of GPU memory, longer time for training and inference, and contains image information not necessarily needed for an assessment of meniscal tears, we decided to crop the data to the Regions of Interest (RoI) of both menisci in an automated pre-processing step that requires segmentations of sufficient quality for training and testing (Tack et al., 2018). Hence, in a second approach (ii), a 3D CNN is trained on these cropped MRIs detecting meniscal tears more accurately than in our first approach. We refer to the second approach as *BB-crop* approach.

We enhanced the performance of our first approach by adding a bounding box regression task. Thus, our final approach (iii) trains a CNN to detect meniscal tears in complete 3D MRI, combined with an additional bounding box regression task leading to an auxiliary loss (the *BB-loss* approach). Framing the problem of meniscal tear detection in this multi-task learning setting – simultaneously solving meniscal tear detection and meniscal bounding box regression – allows our model to implicitly learn to focus on the meniscal regions. Furthermore, segmentation masks are only required during training. Hence, our final approach requires the least data pre-processing at inference time and achieves the best results.

This study presents a method that detects meniscal tears in 3D MRI data on a sub-region level, i.e., the anterior horn, the meniscal body, and the posterior horn for both MM and LM. Formulating the problem in a multi-task learning setting, by adding the information of the location of the menisci as an auxiliary loss to our 3D CNN, state-of-the-art results are achieved. In order to provide an explanation to our CNN’s decision, SmoothGrad saliency maps (Smilkov et al., 2017) are computed and visualized. That way a visual guidance can be given to the clinical domain experts for confirming the results of our approach.

## 2 Materials and Methods

In section 2.1 of this chapter, the data to our method is presented. Thereafter, in sections 2.2 we introduce our data pre-processing and bounding box generation. Section 2.3 is a description of the model architectures utilized in our approach and of their respective components. The particular configuration of our three approaches is illustrated in detail in sections 2.4, 2.5, and 2.6, followed by an explanation of our experimental set-up and training in section 2.7. Finally, a statistical evaluation is summarized in section 2.8 and a method for saliency maps is proposed in section 2.9.

### 2.1 Data from the OAI Database

The publicly available database of the Osteoarthritis Initiative (OAI)^1^ was established to provide researchers with resources to promote the prevention and treatment of knee osteoarthritis. We use 2,399 sagittal Double Echo Steady-State (DESS) 3D MRI scans from the OAI database acquired using Siemens Trio 3.0 Tesla scanners (Peterfy et al., 2006). Additionally, 2,396 sagittal Intermediate-Weighted Turbo Spin-Echo (IW TSE) MRI scans are investigated for the same patients. The demographics of our study are shown in Table 1.

**TABLE 1:** Demographics: In this study, 2,399 DESS and 2,396 IW TSE MRI scans from the OAI database are analyzed. In these data, slightly more normal than diseased medial menisci (MM) and lateral menisci (LM) are contained. Here, normal is defined as no conspicuous features with respect to the MOAKS scoring system in any sub-region.

	DESS	IW TSE
Number of MR images	2,399	2,396
In-plane resolution	0.36 mm × 0.36 mm	0.36 mm × 0.36 mm
Usual slice dimension	384 × 384	442 × 448
Slice thickness	0.7 mm	3 mm
Number of slices	160	35 to 43
Side (left; right)	1104; 1295	1104; 1292
Sex (female; male)	1489; 910	1487; 909
Age [years]	61.88 ± 8.87	61.89 ± 8.86
BMI [kg/m^2^]	29.01 ± 4.79	29.08 ± 4.79
MM (% normal)	60.0%	59.9%
LM (% normal)	80.0%	79.9%

**FIGURE 1:**
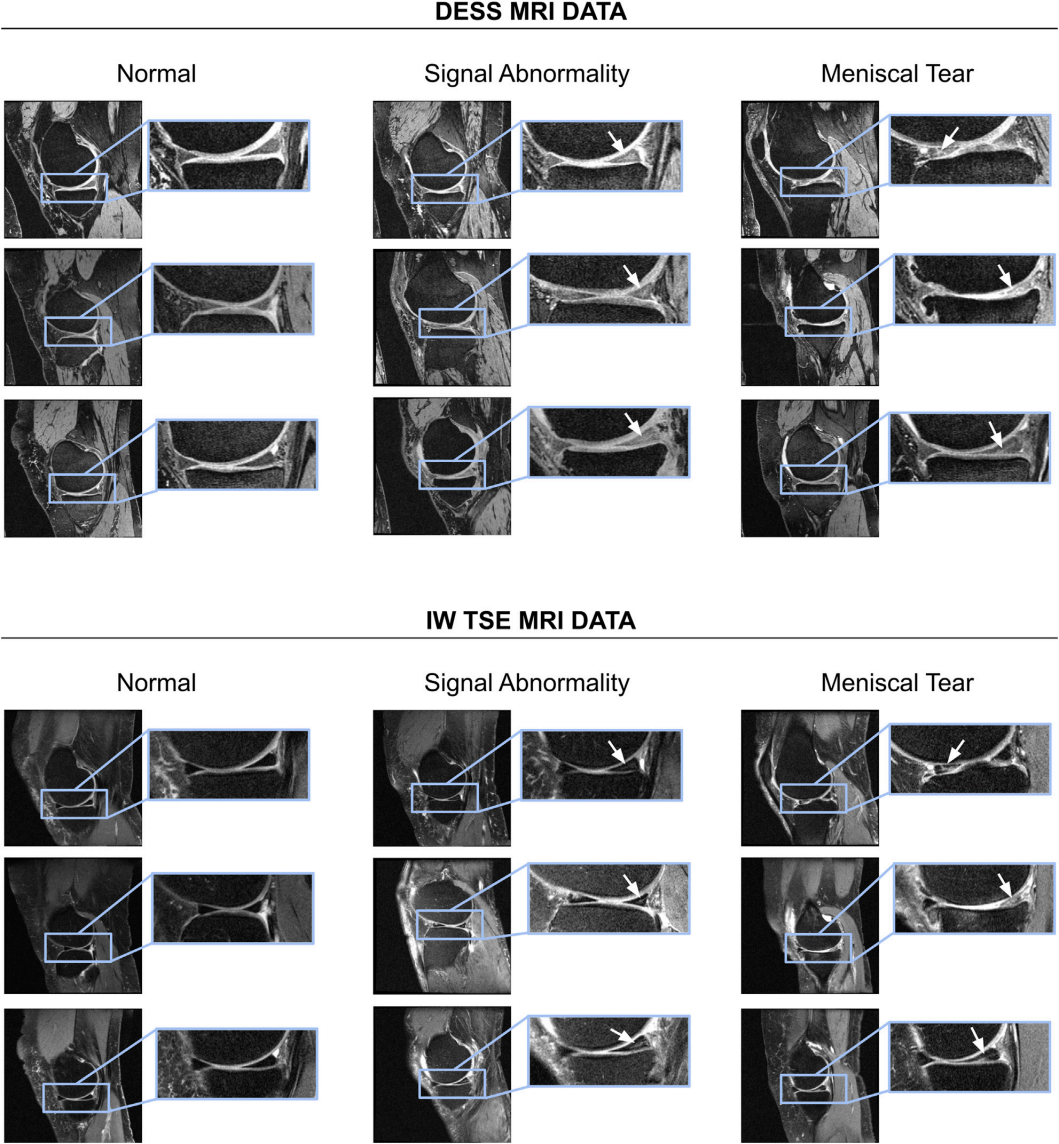
Examples of normal menisci, signal abnormalities, and subjects with meniscal tears shown for DESS as well as IW TSE MRI data. For a summary of different types of meniscal tears per sub-region the reader is referred to Supplementary Table S1.

The OAI database includes multiple reading studies of respective osteoarthritis characteristics, which can be assessed in medical image data. As a gold standard, we utilize labels from MOAKS (Hunter et al., 2011) image reading studies performed by clinical experts. In the MOAKS scoring system, the menisci are divided into three anatomical sub-regions: anterior horn, body, posterior horn. We consider a sub-region as not containing a tear if the MOAKS score is “normal” or indicates a signal abnormality (which is not extending through the meniscal surface and, hence, is no tear). We considered any other type of abnormality (radial, horizontal, vertical, etc.) as a meniscal tear (c.f. Supplementary Table S1). Examples of the MRI sequences, signal abnormalities, and meniscal tears are shown in Figure 1.

### 2.2 Data Pre-processing and Localization of Menisci

**FIGURE 2:**
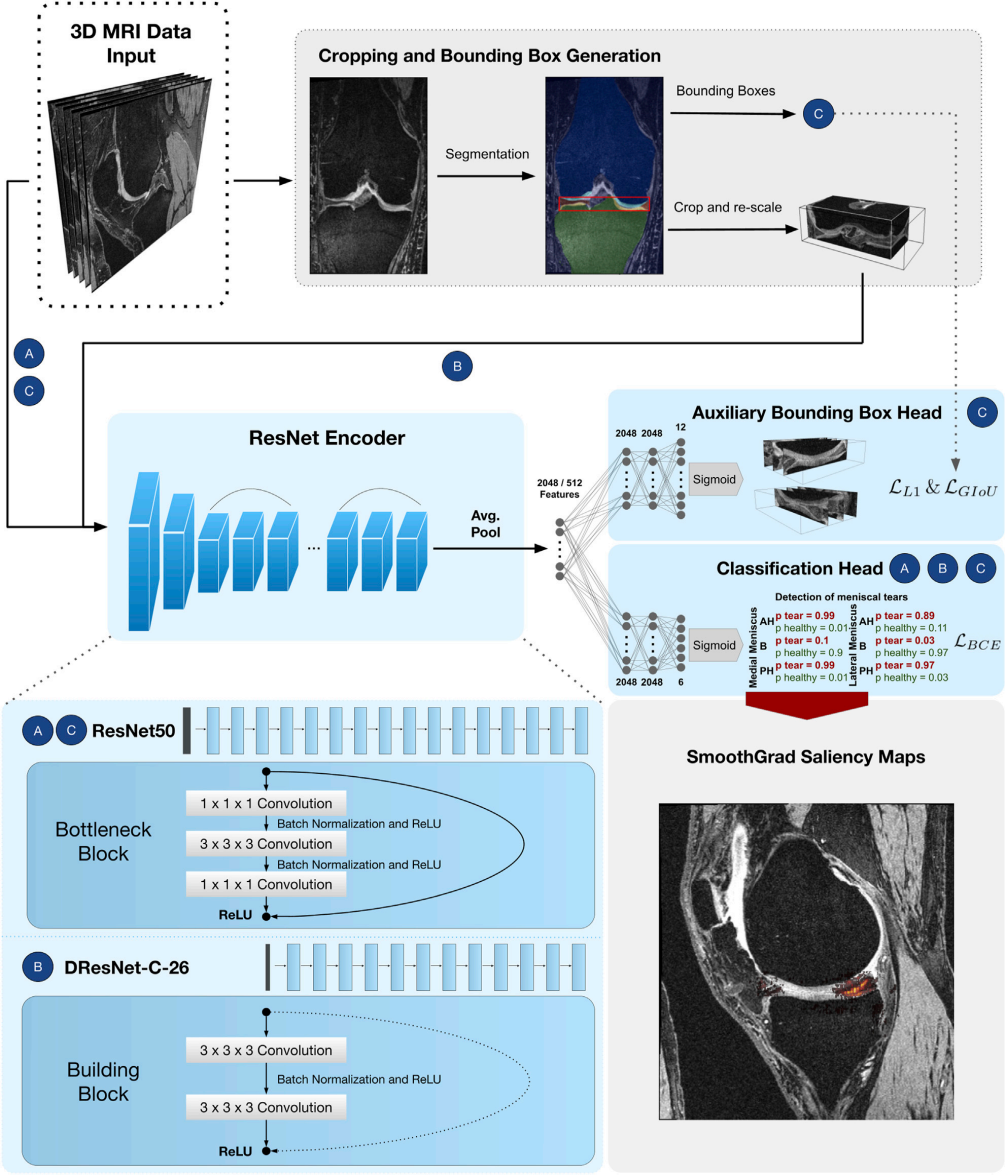
CNN pipeline for detection of meniscal tears in six sub-regions. Approach *Full-scale* uses a ResNet50 encoder followed by a classifier head with $L_{BCE}$ for classification of meniscal tears in 3D MRI data **(A)**. Approach *BB-crop* reduces the 3D MRI input to the meniscal RoI and uses a DRN-C-26 encoder followed by a classifier head with $L_{BCE}$ to detect meniscal tears **(B)**. Approach *BB-loss* uses a ResNet50 encoder followed by a classifier head with $L_{BCE}$ as well as another bounding box regression head with $L_{L1}$ and $L_{GIoU}$ in order to predict bounding boxes of the menisci in the 3D MRI data **(C)**. The ResNet50 is made up of an initial convolutional layer followed by max-pooling before 16 ResNet bottleneck blocks with residual connections are stacked. The DRN-C-26 starts with the same convolutional layer but is immediately followed by ten residual building blocks and, lastly, two building blocks without a residual connection. After average pooling, the encoders generate 2048 and 512 features, respectively. Finally, SmoothGrad saliency maps are presented as overlaid heatmaps on top of the respective MR image to highlight these regions that mostly influenced the detection of tears (bottom right corner).

In a first step of our pre-processing, the intensities of all MR images are scaled to a range of [0, 1] using min-max normalization. Following that, a standardization is applied to each MR image $I_{i}$ according to:

$${\tilde{I}}_{i}=\frac{I_{i}-\mu }{\sigma },$$ (1)

where *μ* is the mean intensity and *σ* is the standard deviation of the training population of normalized scans. Leveraging meniscal segmentations generated by the method of Tack et al. (2018) RoIs spanning the MM and LM are created for DESS MRI data (see Figure 2). RoIs are computed by querying the minimum and maximum position of the menisci along each dimension of the binary segmentation masks: *x*_min_, *x*_max_, *y*_min_, *y*_max_, *z*_min_, *z*_max_. The bounding boxes are uniquely defined as the 3D center coordinate

$$BB_{center}=\left[\begin{array}{c} \left(x_{max}-x_{min}\right)/2+x_{min}\\ \left(y_{max}-y_{min}\right)/2+y_{min}\\ \left(z_{max}-z_{min}\right)/2+z_{min} \end{array}\right]\;,$$ (2)

and with the respective height (*x*_max_ − *x*_min_), width (*y*_max_ − *y*_min_), and depth (*z*_max_ − *z*_min_). These values are represented as relative image coordinates. Hence, a bounding box is defined by 6 floating values: $\left[BB_{center}^{x},BB_{center}^{y},BB_{center}^{z},height,width,depth\right]$ .

For the IW TSE data 600 segmentations are generated in a semi-automated fashion using Amira ZIB Edition^2^ (Reddy, 2017). These masks are defined as voxel-wise annotations of the tissue belonging to the respective meniscus. The method of Tack et al. (2018) was originally developed and evaluated on DESS MRI data. Since the DESS and IW TSE MRI sequences differ significantly in the image resolution (number of slices), that could pose an issue, we have decided to train the self-adapting nnU-net framework (Isensee et al., 2021) on these 600 training datasets. The nnU-net offers 2D and 3D architectures with 3D architectures usually yielding better results (Isensee et al., 2021). For this reason, we have used a 3D variant of the nnU-net that employs 3D convolutions in an encoder-decoder framework with skip-connections. For the IW TSE data, the nnU-net has been automatically configured to have an input size of 24 × 256 × 256 pixels and seven layers of 3D convolutions (Isensee et al., 2021). We train the nnU-net with data augmentation such as random rotations and random cropping using a dice similarity coefficient loss (Isensee et al., 2021) until convergence is reached. Hereby, the dice similarity coefficient is computed between the output of the nnU-net and the respective hand-labelled target segmentation masks. Afterwards, the nnU-net is employed to segment all 2,396 IW TSE MRI scans to yield the respective meniscal RoIs. In order to achieve this, multiple patches of the MRI with a size of 24 × 256 × 256 pixels are being processed by the nnU-net. These patches overlap by half of the patch size in each dimension. Afterwards, the nnU-net framework merges all patches to a final 3D segmentation mask employing a majority voting for every pixel.

### 2.3 Model Architecture

Two distinct models, which are based on 3D counterparts of ResNet architectures (He et al., 2016; Yu et al., 2017) are introduced. ResNets have been widely applied to the medical domain and provide good properties due to the employed skip connections. In theory, the residual connections allow the design of very deep ResNets without exhibiting problems of vanishing gradients (Ide and Kurita, 2017). We have chosen 3D counterparts of 2D ResNets since 3D convolutions are able to comprehend three-dimensional context inherently. It has previously been shown in the context of musculoskeletal MRI analysis that 3D convolutions are more powerful than concatenation of 2D slices as well as a provision of multiple 2D slices as input to a CNN that employs 2D convolutions (Ambellan et al., 2019; Tack and Zachow, 2019). We adapt these 3D ResNet architectures to the three different approaches and their associated input volume sizes. Each model consists of a ResNet encoder followed by one or two Multi-Layer Perceptron (MLP) heads. The *BB-crop* approach has a dilation ResNet-C-26 architecture with an MLP head for the multi-label classification. The *Full-scale* approach has a ResNet50 encoder with a classifier MLP head, and the *BB-loss* approach consists of a ResNet50 encoder with two MLP heads. The performance of the classification task is improved in the BB-loss approach by solving additionally a second task, which is to learn a bounding box regression simultaneously. Again, the first MLP head is employed for multi-label classification. The second MLP head is responsible for the bounding box regression task. All ResNets comprise of a series of convolutional layers, each followed by batch normalization (Ioffe and Szegedy, 2015) and a Rectified Linear Unit (ReLU) activation function (Agarap, 2018).

Our approaches that will be presented in the following sections are designed based on (a selection of) encoders and MLP heads:

#### ResNet50 Encoder

He et al. (2016) proposed a residual layer connection as a way to train deep neural networks without suffering from vanishing gradients. One of their proposed architectures is the ResNet50, with a total of 50 convolutional layers (see Figure 2). The network comprises an initial convolutional layer with kernel size 7 × 7 × 7 followed by a max-pooling layer with kernel size 3 × 3 × 3 and stride 2. The following residual layers are grouped in so-called “bottleneck blocks” (see Figure 2), which are constructed of three convolutional layers. The first and the last are convolutional layers, with kernel size 1 × 1 × 1, where the first one downsamples the number of volume features, and the last one applies feature upsampling. Between these layers, there is a convolutional layer with kernel size 3 × 3 × 3. The bottleneck blocks are arranged in four groups of sizes 3, 4, 6, and 3, where each group starts with a stride of 2 in the first convolutional layer to downsample the feature volumes’ spatial dimensions. Finally, the residual blocks are terminated with a global average pooling (Lin et al., 2013) over the 2048 individual 3D feature volumes coming from the last layer of the ResNet encoder. Computing the average value of each feature map via global average pooling results in a 1D tensor with 2048 features.

#### Dilation ResNet-C-26 Encoder

The DRN-C-26 is a dilated residual CNN architecture with 26 layers introduced by Yu et al. (2017). The original ResNet downsamples the input images by a factor of 32. Downsampling our cropped and uneven sized image volumes by such an amount would result in a loss of information about small and salient parts caused by less expressive feature maps. However, simply reducing the convolutional stride restricts the receptive field of subsequent layers. For this reason, Yu et al. (2017) presented an approach with which downsampling could be reduced while sustaining a sufficiently large receptive field and improving classification results. To construct the DRN-C-26 Yu et al. (2017) applied the following changes to the ResNet18 (He et al., 2016) made of so-called ResNet “building blocks” with two convolutional layers with kernel size 3 × 3 × 3 (see Figure 2). First, the convolutional stride in the last two groups is replaced by dilation. Second, the initial max-pooling layer is replaced by two residual building blocks. Lastly, to reduce aliasing artefacts, a decrease in dilation is added with two final building blocks without residual connections. Again, the residual blocks of the DRN-C-26 are followed by a global average pooling over the 512 feature maps of the last ResNet layer, resulting in a 1D tensor with 512 features.

#### MLP Heads

The features obtained by the respective ResNet encoders are passed through a simple three-layered feed-forward network, also known as MLP, to achieve the respective classifications and regressions. As shown in Figure 2, the MLP input dimension matches the feature dimensions of the CNN (i.e., 2048 neurons in case of ResNet50 and 512 neurons for a DRN-C-26). The hidden layers of all MLP’s consist of 2048 neurons. The classifier head has six output nodes. In the *BB-loss* setting, an additional three-layered MLP with twelve output nodes was added to perform a bounding box regression.

### 2.4 *Full-Scale* Approach: Detection of Meniscal Tears in Complete MRI Scans

In our first and most straightforward approach, the complete 3D MRI is provided as input to the CNN. The CNN consists of a ResNet50 encoder followed by an MLP head. The outputs of the MLP after a sigmoid activation represent the probabilities for the six meniscal sub-regions to contain a tear.

The CNN is trained by minimizing the binary cross-entropy loss $L_{BCE}$ for a given batch of *N* samples. With a target matrix $Y\in Z_{2}^{N\times C}$ and an output matrix $\hat{Y}\in R^{N\times C}$ for all *C* meniscal sub-region labels the definition of $L_{BCE}$ is:

$$L_{BCE}=\frac{1}{N}\overset{C}{\underset{c=1}{\sum }}\overset{N}{\underset{i=1}{\sum }}w_{c}\left[y_{i,c}log\left(\sigma \left({\hat{y}}_{i,c}\right)\right)+\left(1-y_{i,c}\right)log\left(1-\sigma \left({\hat{y}}_{i,c}\right)\right)\right],$$ (3)

where *w*_*c*_ is an inverse weighting of label frequencies and *σ*(⋅) is a sigmoid activation function. The *Full-scale* approach is visualized under A) in Figure 2.

### 2.5 *BB-Crop* Approach: Detection of Meniscal Tears in Cropped MRI Datasets

Cropping 3D MRI data to the meniscal RoI is expected to provide two desirable properties. First, it provides smaller volumes reducing the required GPU memory as well as the run time. Second, the *Full-scale* 3D MR images can be considered noisy as they provide additional and unnecessary information about surrounding anatomical structures. By cropping the data to the RoI of the menisci, this unnecessary information is suppressed. Leveraging the RoI generated as described in section 2.2 the 3D MR images are cropped with a 5% margin around the menisci. Each cropped image is then resampled with trilinear interpolation to the closest multiples of 16, given the biggest bounding box in the training set. Figure 2 visualizes the cropping and resampling process. Consequently, the cropped and resampled images have a size of (64, 64, 176) for the DESS data and (16, 64, 176) for the IW TSE data. *BB-crop* utilizes a Dilation Resnet-C-26 encoder followed by an MLP classifier head. The CNN is trained by minimizing the $L_{BCE}$ as given in Eq. 3. The framework is visualized under B) in Figure 2.

### 2.6 *BB-Loss* Approach: Detection of Meniscal Tears in Complete MRI Scans Enhanced by Regression of Meniscal Bounding Boxes

The *BB-crop* approach requires segmentation of both menisci (or at least the determination of a meniscal region) in training and testing. Since generating segmentations is time-consuming (the method of Tack et al. (2018) requires approximately 5 min of run time), it is beneficial to avoid this step. Moreover, this approach heavily relies on high-quality bounding boxes in training and inference, which are difficult to obtain and strongly influence the performance quality. Thus, the motivation for our final *BB-loss* approach is to detect meniscal tears in 3D MRI data without extensive pre-processing requirements such as segmenting the menisci or computing bounding boxes for meniscal regions. Instead, the location of the menisci is added as an additional loss term for the training. The encoder is kept identical to the *Full-scale* approach, namely a ResNet-50 encoder. Furthermore, an identical MLP head is utilized for the meniscal tear detection. Additionally, we show that the meniscal position information helps the CNN to focus on these regions in the image yielding better results. A second MLP head is employed in the *BB-loss* approach to regress the coordinates of the meniscal RoI. By incorporating this knowledge as a loss in the training process, the locations of the menisci must not be explicitly provided at test time. The total loss in the *BB-loss* setting is computed considering the multi-label classification and the bounding box regression task. For detection of meniscal tears $L_{BCE}$ is employed (Eq. 3). In the bounding box regression the outputs of the MLP head are 6 coordinates *d* for the MM and LM, respectively. Utilizing a sigmoid activation function, these values are given as relative positions within the image in a range of [0, 1] of the respective dimension. For a detailed description of the bounding box generation procedure, we refer the reader to section 2.2. The first component of the bounding box loss is an L1-term $L_{L1}$ defined as

$$L_{L1}=\|B-\hat{B}\|,$$ (4)

with a predicted bounding box $\hat{B}$ and a target bounding box *B* that is derived from the automated segmentation masks. These *N* × 2*d* matrices contain *N* rows with medial and lateral bounding box values. Where *b*_*n*,*i*_ and ${\hat{b}}_{n,i}$ describe the *n*th element of the batch and the *i*th value of the concatenated bounding boxes. With this formulation the loss is given as

$$L_{L1}=\frac{1}{N}\overset{N}{\underset{n=1}{\sum }}\overset{2d}{\underset{i=1}{\sum }}|b_{n,i}-{\hat{b}}_{n,i}|.$$ (5)

The second component of the bounding box loss is a modified Intersection over Union (IoU) term, more specifically the Generalized-IoU (GIoU) $L_{GIoU}$ (Rezatofighi et al., 2019) defined as:

$$L_{GIoU}=1-IoU+\frac{\left|C\backslash \backslash \left(B\cup \hat{B}\right)\right|}{\left|C\right|},$$ (6)

where C is a convex hull enclosing the predicted and the target box. The operator |⋅| computes the box volume. The convex hull is the smallest possible region that encloses both the output and the target bounding boxes. It can be defined as a bounding box, fully characterised by the 6 coordinates elaborated above. It is computed by taking the minimum and maximum extent of both the target bounding box and the predicted bounding box coordinates along the x-, y- and z-axis. The numerator of the third term of the $L_{GIoU}$ is the convex hull volume subtracted by the volume of *B* and $\hat{B}$ , and the denominator is the volume of the convex hull. Hence, the third term of the $L_{GIoU}$ can be considered as the relative volume of the convex hull not covered by the union of predicted and target bounding box. The IoU is defined as $\frac{\left|B\cap \hat{B}\right|}{\left|B\cup \hat{B}\right|}$ , that is, the ratio of the intersecting voxels of *B* and $\hat{B}$ to their union. The $L_{GIoU}$ is computed for each meniscal RoI and averaged for the given batch. The overall loss L for the *BB-loss* approach is given as

$$L=L_{BCE}+L_{L1}+L_{GIoU}.$$ (7)

The *BB-loss* approach is visualized under C) in Figure 2.

### 2.7 Experimental Setup and Training of CNNs

The given MRI data of the OAI are randomly split into 50% training data, 15% validation data and 35% testing data. Hence, our two experiments have 1200/359/840 and 1197/359/840 training/validation/testing scans for the DESS data and the IW TSE data, respectively. We implemented the CNNs of all approaches in PyTorch 1.9. Convolutional weights are initialized using a normal distribution as in He et al. (2015) tailored towards our deep neural networks with asymmetric ReLU activation functions. While, batch normalization weights and biases are initialized constant with 1 and 0. We train our CNNs on an Nvidia A100 GPU with 40 GB memory. Training our three ResNets, separate learning rates and dropout probabilities for the ResNet-encoders and the MLP-heads are introduced. Suitable learning rate, dropout and batch size hyper-parameters are found using the validation data of the DESS scans. The learning rate values for all parts (ResNet encoder, classifier head and bounding box head) are evaluated in an interval of [ 1e−5 , 0.01]. Dropout percentages are varied in an interval of [0.1, 0.9]. Further, the training batch size limited by the input size is varied from 2 to 64 for the *BB-crop* approach. Due to a larger input volume in approach *Full-scale* and *BB-loss*  batch size was kept constant at a value of 4. For a complete summary of our hyper-parameter values, please refer to Supplementary Table S2. Training is performed using the ADAM optimizer (Kingma and Ba, 2014) with *β*_1_ = 0.9, *β*_2_ = 0.999 and *ϵ* = 1e−08 with a learning rate decay of 0.5 every 50 epochs. Training on the IW TSE sequence is not performed from scratch, instead, both ResNet encoder and MLP weights are fine-tuned. In both DRN-C-26 and ResNet50 cases, we use the CNNs that achieve the lowest validation loss on the DESS sequence.

On-the-fly data augmentation is performed during training. Specifically, this means, random cropping around the RoI, horizontal flips, rotations, Gaussian noise, and intensity scaling are applied with 50% probability. For the *Full-scale* approach, we perform random cropping of up to 10% along coronal, 20% sagittal and 20% axial direction. In the *BB-crop* approach, random crops are performed by uniformly cropping within a 20*%* margin around the menisci. The *BB-loss* approach uniformly samples possible crops around the menisci. All cropped images are resampled with trilinear interpolation to attain consistent sizes per approach and dataset. Input images for the *Full-scale* and *BB-loss* approach are sampled for the DESS sequence data to (160, 384, 384) and for IW TSE images to (44, 448, 448). The *BB-crop* approach resamples to (64, 64, 176) and (16, 64, 176), respectively. The added Gaussian noise is pixel-wise sampled as ϵ∈N(0.1,0.5) . The random rotation is uniformly sampled from U(−5°,+5°) and image intensity is scaled by a uniformly sampled multiplication factor b∈U(0.9,1.1) .

### 2.8 Statistical Assessment of Detection Quality

For all experiments, we plot the true positive rate (TPR = sensitivity) against the false positive rate (FPR = 1–specificity) at various decision thresholds to create ROC curves (Brown and Davis, 2006). Additionally, we compute the ROC AUC to assess the quality of our classifiers. The quality of our predicted bounding boxes is assessed by computing the IoU with the target bounding boxes. We consider IoU values over 0.5 as successful localization of the menisci since this is a common value in object detection tasks (Girshick et al., 2014).

### 2.9 SmoothGrad Saliency Map Visualizations for Areas Addressed by the CNN

Gradient saliency maps (Simonyan et al., 2013) (otherwise called pixel attribution maps or sensitivity maps) highlight pixel regions in the input image that mostly influenced a neural network’s decision. To attain such pixel attributions, one computes the derivative of the final linear layer in a neural network with respect to the input via back-propagation. More formally, a gradient saliency map *S*_*c*_ for a sub-region c for which our neural network *f* yields a detection of meniscal tears is calculated as:

$$S_{c}\left({\tilde{I}}_{i}\right)=\frac{\partial f_{c}\left({\tilde{I}}_{i}\right)}{\partial {\tilde{I}}_{i}}.$$ (8)

For our two most promising approaches *BB-crop* and *BB-loss*, these maps are computed by applying a slight enhancement to the original mechanism - the SmoothGrad method (Smilkov et al., 2017). Similar to the SmoothGrad approach of Smilkov et al. (2017) we augmented the input image slightly, introducing noise, such that through averaging, the saliency maps of different noise levels are smoothed out. We apply Gaussian distributed noise ϵ∈N(0.1,0.5) , random horizontal flips, uniformly sampled rotations r∈U(−5°,+5°) and uniformly sampled pixel intensity shift with a multiplication factor b∈U(0.9,1.1) . Each image is augmented 20 times with a probability of 50*%* per augmentation, and the resulting maps are averaged.

## 3 Results

We applied all approaches to DESS as well as IW TSE data from the OAI database. Each of our approaches detects meniscal tears for the MM and the LM. In particular, tears are detected in the three anatomical sub-regions anterior horn, meniscal body, and posterior horn. All results are presented in this section.

### 3.1 Detection of Meniscal Tears in DESS MRI Data

**FIGURE 3:**
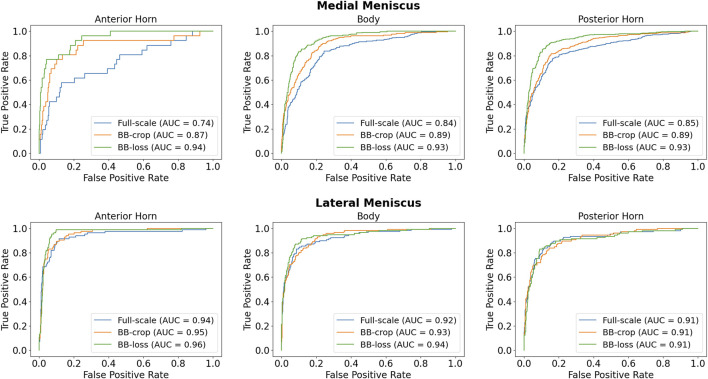
ROC curves for detection of meniscal tears in DESS MRI data.

Employing the *Full-scale* approach, the AUC values are 0.74, 0.84, 0.85 for the anterior horn, body, and posterior horn of the MM. For the LM, the AUC values are 0.94, 0.92, 0.91. The *BB-crop* approach usually yields higher AUC values, being 0.87, 0.89, 0.89 and 0.95, 0.93, 0.91. The *BB-loss* gives the highest AUC values, being 0.94, 0.93, 0.93 and 0.96, 0.94, 0.91. The ROC curves employing all three approaches are shown in Figure 3. In addition, all ROC AUC results are summarized in Table 2.

**TABLE 2:** ROC AUC results for medial menisci (MM) and lateral menisci (LM) in DESS MRI data.

	MM			LM		
Method	Anterior	Body	Posterior	Anterior	Body	Posterior
*Full-scale*	0.74	0.84	0.85	0.94	0.92	0.91
*BB-crop*	0.87	0.89	0.89	0.95	0.93	**0.91**
*BB-loss*	**0.94**	**0.93**	**0.93**	**0.96**	**0.94**	**0.91**

The best results for each anatomical sub-region are highlighted in bold.

### 3.2 Detection of Meniscal Tears in IW TSE MRI Data

**FIGURE 4:**
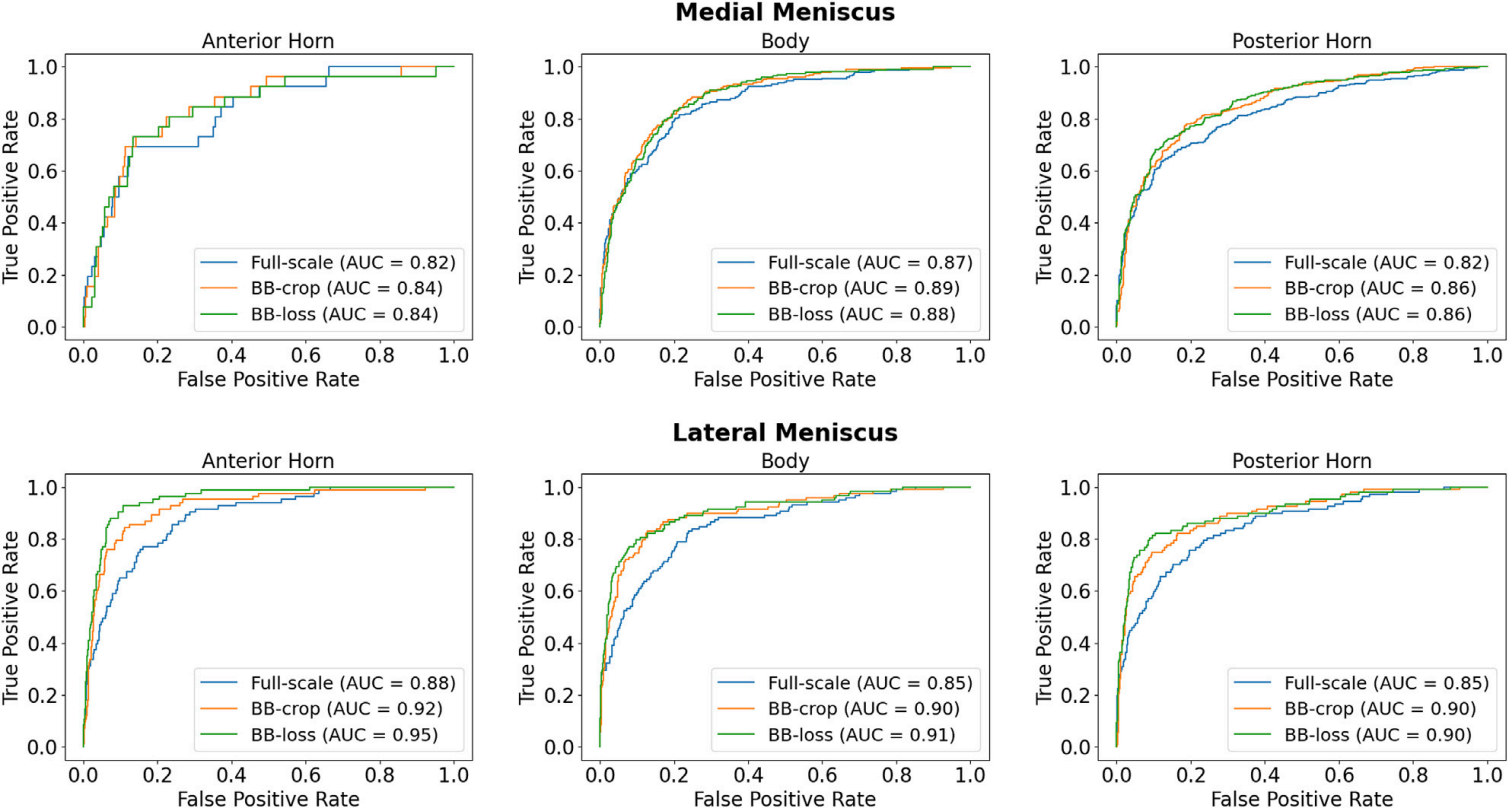
ROC curves for detection of meniscal tears in IW TSE MRI data.

Employing the *Full-scale* approach, the AUC values are 0.82, 0.87, 0.82 for the anterior horn, body, and posterior horn of the MM. For the LM, the AUC values are 0.88, 0.85, 0.85. The *BB-crop* approach usually yields higher AUC values, being 0.84, 0.89, 0.86, and 0.92, 0.90, 0.90. The *BB-loss* gives similar AUC values, being 0.84, 0.88, 0.86, and 0.95, 0.91, 0.90. The ROC curves of all approaches are shown in Figure 4. Further, all AUC values are summarized in Table 3.

**TABLE 3:** ROC AUC results for medial menisci (MM) and lateral menisci (LM) in IW TSE data.

	MM			LM		
Method	Anterior	Body	Posterior	Anterior	Body	Posterior
*Full-scale*	0.82	0.87	0.82	0.88	0.85	0.85
*BB-crop*	**0.84**	**0.89**	**0.86**	0.92	0.90	**0.90**
*BB-loss*	**0.84**	0.88	**0.86**	**0.95**	**0.91**	**0.90**

The best results for each anatomical sub-region are highlighted in bold.

### 3.3 Localization of Menisci via the *BB-Loss* Approach

**FIGURE 5:**
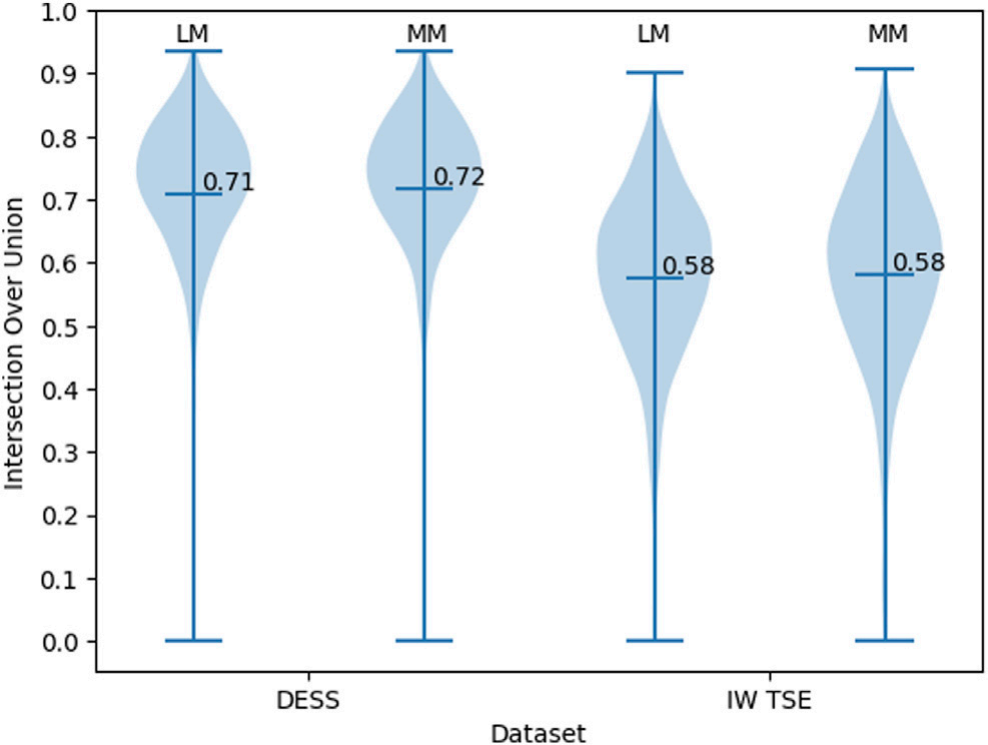
The distribution of the IoU values for the bounding boxes of MM and LM in DESS and IW TSE MRI data.

To investigate the bounding box regression quality of the proposed method we evaluate the distribution of the IoU values for the predicted bounding boxes (Figure 5). For the DESS dataset (our primary benchmark), we observed a very high quality of MM and LM bounding box predictions. With the values being close to normally distributed around a mean value of 0.71 (95% confidence interval (CI): 0.71–0.72) and standard deviation of 0.13. With the IoU threshold of 0.5, we conclude that 95% of the resulted bounding boxes are identified correctly. Unfortunately, we observed a clear decrease in the object detection performance in the IW TSE dataset. With a mean value of 0.58 (95% CI: 0.57–0.59) and a standard deviation of 0.14. Applying the same detection threshold as above we testify, that only around 76% of menisci were detected correctly, with the overall quality of the bounding boxes being more widely spread.

### 3.4 Visualization of Areas Addressed by the CNN

**FIGURE 6:**
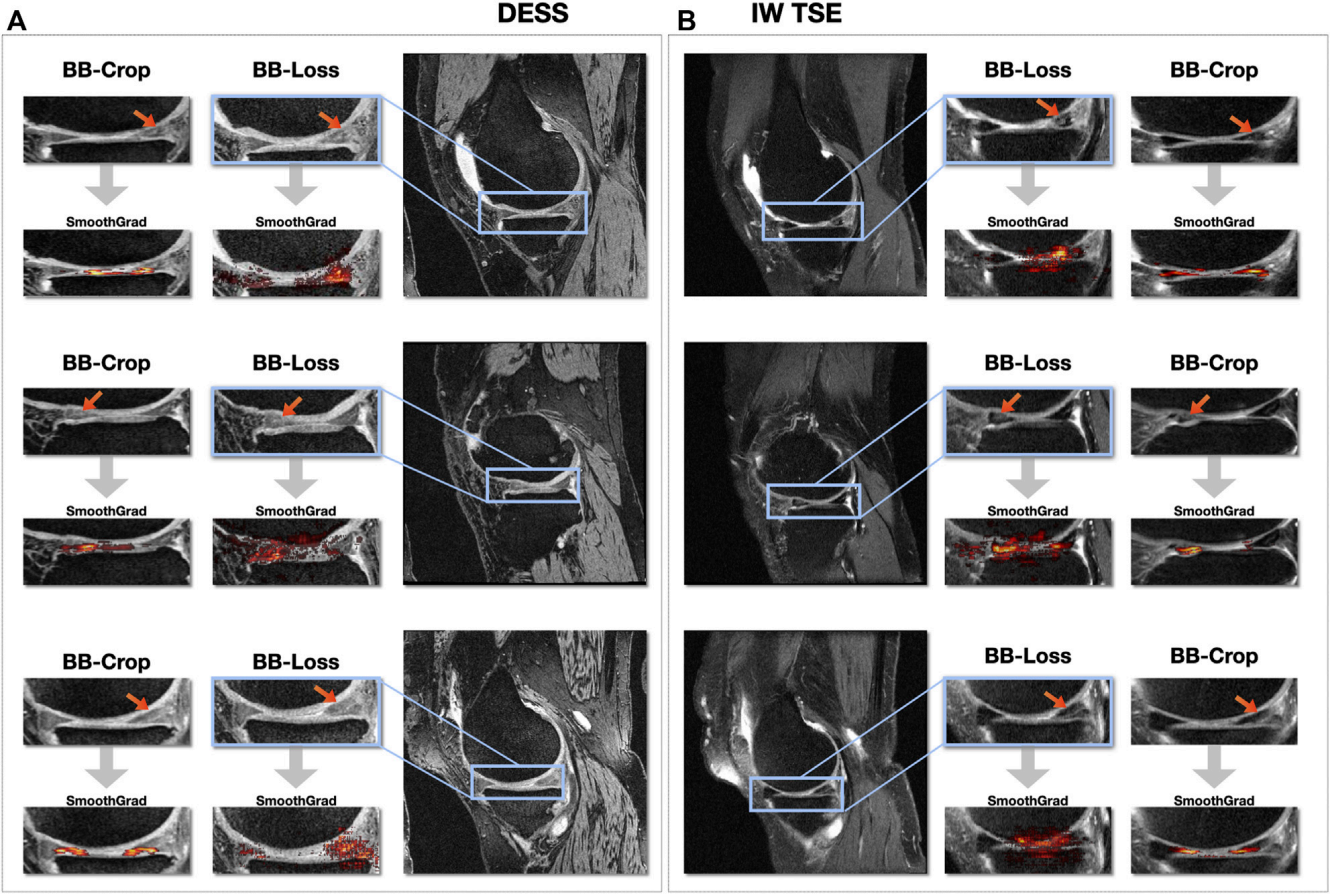
SmoothGrad saliency maps overlaid over DESS MRI data **(A)** and IW TSE MRI data **(B)**.

Figure 6 shows SmoothGrad saliency maps for the *BB-crop* and *BB-loss* approach overlaid to MR images. Examples are shown for randomly selected test cases, displaying different kinds of meniscal tears for DESS and IW TSE data. The RoIs for the *BB-loss* approach were extracted using predicted bounding boxes and the respective close-ups are shown. Red arrows point at the location of meniscal defects. Most saliency maps obtained this way display a plausible localization of the meniscal tears. The plausibility of these maps was qualitatively evaluated by their correspondence to the target labels of the regions in which the tears could also be confirmed with the help of visual inspection of the image data. SmoothGrad saliency maps are capable of highlighting more than just one affected sub-region, i.e., in the presence of defects in multiple sub-regions of one meniscus, one similarly observes these being correctly highlighted. With the Dilation ResNet-C-26 employed in the *BB-crop* approach, we observed that this CNN yields smoother and less noisy SmoothGrad saliency maps. However, in many cases, ResNet-50 saliency maps targeted the affected region better, but did not outline this region sharply.

### 3.5 Detection Performance—Different Sub-regions and Defect Types

Even though the occurrence of defects varies between meniscal sub-regions (see Supplementary Figure S2), we observe only minimal differences between AUC values of sub-regions in DESS MRI data (c.f. Table 2). However, we analyzed the false positive classifications and found that for all sub-regions, signal abnormalities were more often misclassified than normal menisci were (see Supplementary Figure S1). The misclassification rate of signal abnormalities is highest for the posterior horn of the lateral meniscus, the region with the least AUC for the DESS data. Conversely, the lowest signal abnormality misclassification rate is prevalent in the posterior horn of the medial meniscus, the sub-region with the highest number of signal abnormalities (Supplementary Table S1).

The least common types of tears occurring in the data are radial and vertical tears, amounting to 72 and 69, respectively. Vertical tears were most challenging for our method to detect in DESS data and led to the most false negative results (see Supplementary Figure S2). Radial meniscal tears were the ones yielding the second highest rate of misclassifications.

## 4 Discussion

The primary goal of our work was to develop a method that provides an efficient, robust and automated way to detect and better locate meniscal tears in MRI data, that is, the detection of tears with respect to the anatomical regions in which they occur. We devised a procedure that utilizes a 3D CNN to process arbitrary 3D MRI data without the need for any extensive pre-processing.

Many previously proposed methods already yield a high accuracy in the detection of meniscal tears. To compare our results to the related work, we focus our assessment on the results of our *BB-loss* approach on the DESS MRI data. Our method detects meniscal tears in anatomical sub-regions of MM and LM. However, it has not been explicitly trained for menisci tear detection in the entire knee as well as the two menisci. Therefore, to obtain the respected values, we performed max operations on our CNNs’ outputs. A comparison of the different approaches with their respective detection AUC is summarized in Table 4. Our *BB-loss* approach achieved state-of-the-art results in detecting meniscal tears in the medial and lateral meniscus with an AUC of 0.94 and 0.93. For the task of meniscal tear detection in the entire knee *BB-loss* approach had an AUC of 0.94 is second to the approach of Fritz et al. (2020). However, the proposed methods from the related work still leave a desire for a more precise spatial assignment of the findings. For instance, localizing tears per meniscus or in anatomical sub-regions thereof. For tear detection per meniscus, our method performs better than related work (Fritz et al., 2020; Rizk et al., 2021). However, the novelty of our method is the detection of tears for each anatomical sub-region of the menisci in 3D MRI data, providing an anatomically more detailed localization.

**TABLE 4:** Comparison of our results on DESS MRI data to the related work. The “3D data” column indicates whether the method is trained on and applied to complete 3D MR images. The explainable AI “XAI” column indicates if concepts of saliency maps are employed in order to highlight the areas responsible for the CNNs’ decisions.

	Roblot et al. (2019)*	Couteaux et al. (2019)*	Bien et al. (2018)	Pedoia et al. (2019)	Tsai et al. (2020)	Azcona et al. (2020)	Fritz et al. (2020)	Rizk et al. (2021)	Ours: *Full-scale*	Ours: *BB-crop*	Ours: *BB-loss*
3D data	×	×	*✓*	*✓*	*✓*	*✓*	*✓*	*✓*	*✓*	*✓*	*✓*
XAI	×	*✓*	*✓*	×	*✓*	×	*✓*	*✓*	*✓*	*✓*	*✓*
Anywhere	0.94	0.906	0.847	0.89	0.904 and 0.913	0.934	**0.961**	—	0.81	0.89	0.94
Any MM	—	—	—	—	—	—	0.882	0.93	0.79	0.89	**0.94**
Any LM	—	—	—	—	—	—	0.781	0.84	0.87	0.92	**0.93**
MM-AH	*✓*	*✓*	—	—	—	—	—	—	0.85	0.84	**0.94**
MM-B	—	—	—	—	—	—	—	—	0.82	0.89	**0.93**
MM-PH	*✓*	*✓*	—	—	—	—	—	—	0.78	0.89	**0.93**
LM-AH	*✓*	*✓*	—	—	—	—	—	—	0.90	0.95	**0.96**
LM-B	—	—	—	—	—	—	—	—	0.86	0.92	**0.94**
LM-PH	*✓*	*✓*	—	—	—	—	—	—	0.88	**0.91**	**0.91**

*Roblot et al. (2019) and Couteaux et al. (2019) detected meniscal tears in 2D slices for AH and PH, but reported overall results only. The best methods for the respective task are highlighted in bold.

With AUC values being consistently higher than 0.90 for DESS MRI data, our approach achieves excellent detection quality for all meniscal sub-regions using uncropped 3D MRI volumes. We also show that our method generalizes well to other MRI sequences, that is, from DESS to IW TSE data. IW TSE data provides a more challenging setting with a higher slice thickness in the mediolateral direction. Moreover, for certain meniscal defects, such as horizontal tears in the meniscal body, a lower resolution in the acquired MR image direction significantly reduces the visibility of the features required for an accurate classification. The result could be improved by using an input image with an isotropic resolution. Such an image can be obtained by either upsampling an existing image or, even better—acquiring a new image, at a higher resolution.

Signal abnormalities are still a challenge. In cases where menisci with tears are to be distinguished from menisci without tears, signal abnormalities are currently regarded as the latter. A fine-grained differentiation between tears and signal abnormalities is likewise a challenge to our method, primarily through the ambiguous image appearance. Potentially, more training data, as present for the region with the most signal abnormalities—the MM posterior horn, would allow our CNN to better learn to distinguish signal abnormalities from tears.

We expected our model to generalize to all meniscal pathologies but observed problems detecting vertical and radial tears. However, these tears were less common in the available training data, and we believe that more data on such cases would enable our method to detect vertical and radial tears with higher accuracy. Furthermore, coronal and axial imaging sequence orientation could provide additional insights (Bien et al., 2018), possibly improving the detection of otherwise barely visible tears.

One major limitation that we see is that our method still requires a localization of the menisci in training. However, other segmentation approaches or (non-automatic) approaches could be applied to attain bounding boxes, possibly improving results by providing more accurate bounding boxes for training.

## 5 Conclusions and Future Work

We present a method in an efficient and fully automated multi-task learning setting that accurately detects meniscal tears on a sub-region level in MM and LM. Our method yields the best results on sagittal DESS MRI data and generalizes well to sagittal IW TSE data. Further, visual support for clinical detection of meniscal tears is provided by SmoothGrad saliency maps highlighting regions that mainly contributed to the decision.

Future work could comprise an analysis of anomaly detection (normal vs. signal abnormality vs. torn menisci) or a classification of different types of tears (horizontal, radial, complex, etc.). Since some of these types occur only rarely for specific sub-regions, deep learning-based methods probably require a lot more image data or data generated with generative models. Also, new issues of class imbalances will arise for the classification of tear types.

From the method perspective, the choice of an encoder provides opportunities for improvement. For instance, recent self-attention mechanisms, so-called “transformer” architectures (Vaswani et al., 2017; Dosovitskiy et al., 2020) are worth an investigation. Since transformers typically require a vast amount of training data, they might not necessarily lead to better accuracy, but the self-attention maps (Caron et al., 2021) may result in a more meaningful explanatory power than classical methods of saliency mapping. Also, generative adversarial networks have been recently employed for explaining the decision of CNN’s (Katzmann et al., 2021; Shih et al., 2021). As deep learning methods become more precise in localizing meniscal tears coupled with further sophisticated concepts on explainability, CAD tools will become practical for clinical decision support. In future work, we plan to investigate whether our method better assists physicians in their diagnostic tasks.

## Data Availability

Publicly available datasets were analyzed in this study. This data can be found here: https://nda.nih.gov/oai/ for the MR images analyzed in this study as well as the medical image annotations from the NIH OAI archive. The employed segmentation masks of all MM and LM will be made publicly available at https://pubdata.zib.de upon publication of this paper.

## Footnotes

1
https://nda.nih.gov/oai/

2
https://amira.zib.de
