# Supplementary material for: A Multi-Task Deep Learning Method for Detection of Meniscal Tears in MRI Data from the Osteoarthritis Initiative Database
Source: Front Bioeng Biotechnol. 2021 Dec 2;9:747217. doi: 10.3389/fbioe.2021.747217 (PMC8675251; doi:10.3389/fbioe.2021.747217)
Supplement: Supplementary file 1 [file DataSheet2.PDF]

Supplement 2

Table S2: Meniscal tear type frequencies across meniscal sub-regions for the DESS and IW TSE sequence.

Meniscal morphology per sub-region (DESS)

Type	MM-AH	MM-B	MM-PH	LM-AH	LM-B	LM-PH
Normal	2,109	1,241	545	1,819	1,721	1,780
Signal Abnormality	209	576	971	301	317	327
Radial Tear	2	5	33	6	8	18
Horizontal Tear	17	206	476	121	207	143
Vertical Tear	1	11	30	6	11	10
Complex Tear	0	13	44	4	9	11
Partial Maceration	54	345	294	105	123	104
Complete Maceration	5	2	4	34	3	2
NaN	2	0	2	3	0	4

Meniscal morphology per sub-region (IW TSE)

Type	MM-AH	MM-B	MM-PH	LM-AH	LM-B	LM-PH
Normal	2,107	1,238	544	1,818	1,718	1,778
Signal Abnormality	209	576	971	300	317	327
Radial Tear	2	5	33	6	8	18
Horizontal Tear	17	206	475	121	207	143
Vertical Tear	1	11	30	6	11	10
Complex Tear	0	13	44	4	9	11
Partial Maceration	54	345	294	105	123	104
Complete Maceration	5	2	4	34	3	2
NaN	1	0	1	2	0	3
