# Supplementary figures and images for: A Multi-Task Deep Learning Method for Detection of Meniscal Tears in MRI Data from the Osteoarthritis Initiative Database
Source: Front Bioeng Biotechnol. 2021 Dec 2;9:747217. doi: 10.3389/fbioe.2021.747217 (PMC8675251; doi:10.3389/fbioe.2021.747217)

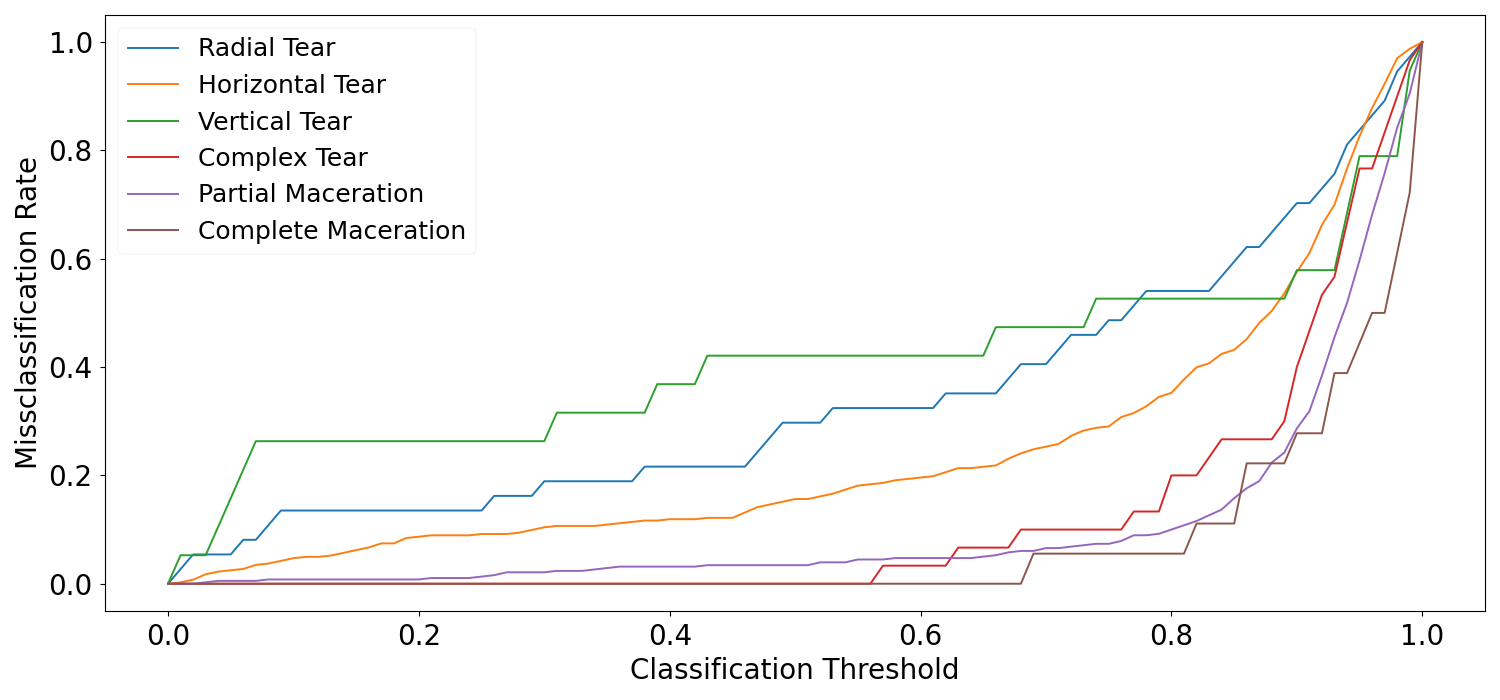

Supplement: Supplementary file 2 [file Image1.tiff]

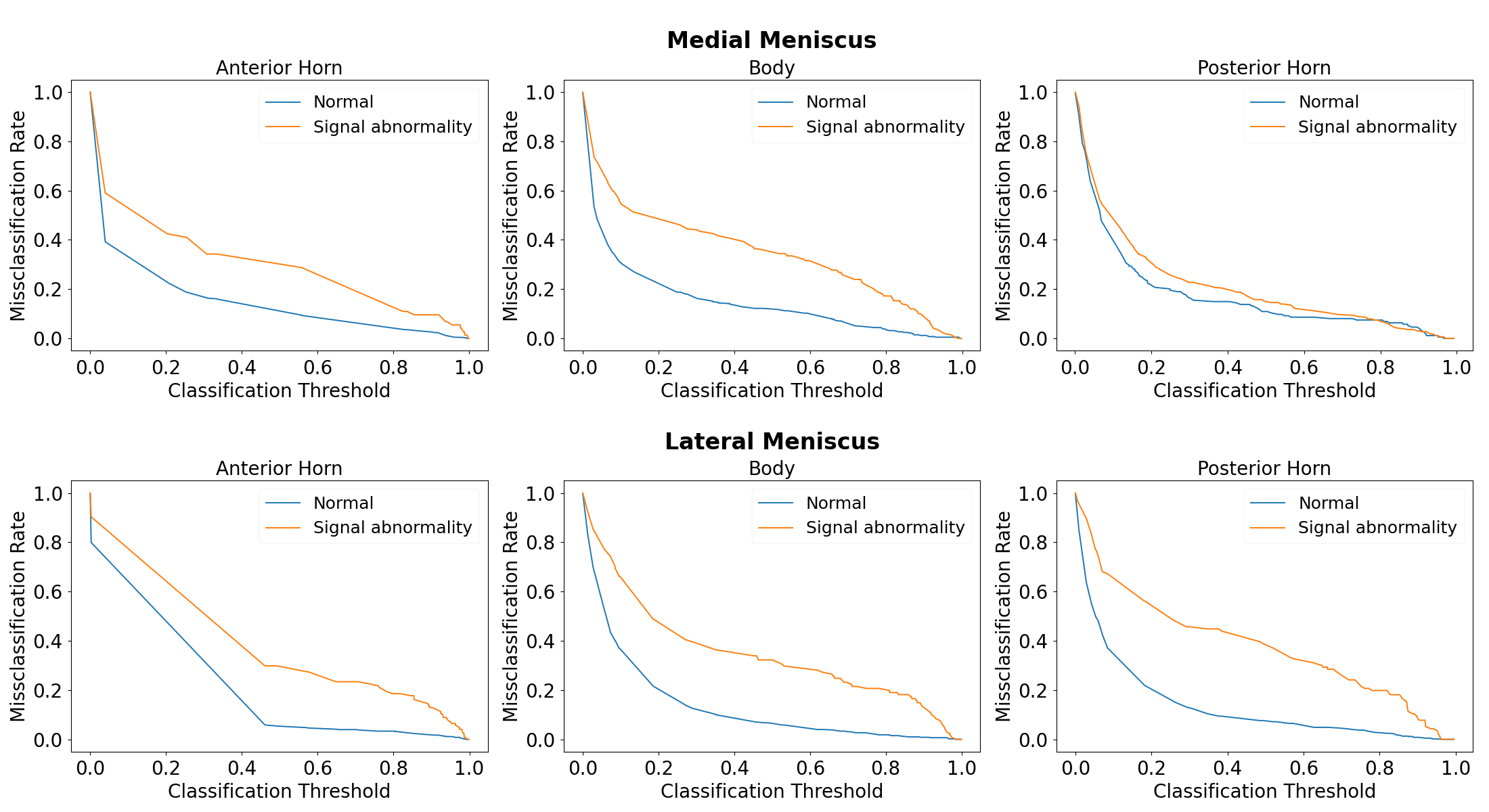

Supplement: Supplementary file 4 [file Image2.tiff]
