# Supplementary material for: A Multi-Task Deep Learning Method for Detection of Meniscal Tears in MRI Data from the Osteoarthritis Initiative Database
Source: Front Bioeng Biotechnol. 2021 Dec 2;9:747217. doi: 10.3389/fbioe.2021.747217 (PMC8675251; doi:10.3389/fbioe.2021.747217)
Supplement: Supplementary file 3 [file DataSheet1.PDF]

Supplement 1

Table S1: Hyper-parameters of our methods. This table shows the learning rate of the respective encoder (LR-ENC) and MLP head (LR-MLP). Also, the dropout probabilities of the respective encoder (DO-ENC) and MLP head (DO-MLP) are provided. Finally, the batch size (BS) used for training the method is given. The same set of hyper-parameters was used for the DESS as well as IW TSE dataset.

Method	LR-ENC	LR-MLP	DO-ENC	DO-MLP	BS
Full-scale	5.0e-4	5.0e-5	50%	10%	4
BB-crop	4.4e-4	6.1e-5	76%	19%	13
BB-loss	5.0e-4	5.0e-5	50%	10%	4
